# An update on the genetics of alcoholic liver disease

**DOI:** 10.3389/fgstr.2022.1030399

**Published:** 2022-11-30

**Authors:** Ravikanth Vishnubhotla, Anand V. Kulkarni, Mithun Sharma, Padaki Nagaraja Rao, Duvvur Nageshwar Reddy

**Affiliations:** ^1^ Asian Healthcare Foundation (A research wing of AIG Hospitals), Mindspace Road, Gachibowli, Hyderabad, Telangana, India; ^2^ AIG Hospitals, Mindspace Road, Gachibowli, Hyderabad, Telangana, India

**Keywords:** Alcohol-related liver disease, susceptibility, genetics, variants, *PNPLA3*, *TM6SF2*

## Abstract

Worldwide, an estimated 2 billion individuals consume alcohol, which contributes to short-term or long-term consequences on health and social life. Alcohol is the cause of approximately 1.8 million deaths per year, representing 3.2% of all deaths worldwide. Of the 2 billion individuals who consume alcohol, more than 75 million are diagnosed with alcohol-use disorder (AUD) and are at an enhanced risk of developing alcoholic liver disease (ALD). However, not all individuals who consume alcohol develop liver disease suggesting the intricate interactions of host genetics with the environment in the precipitation of the phenotype. With advances in genomic technologies, it is now possible to sequence clinically relevant genomic loci associated with a phenotype with precision and faster turnaround times. Genomic data in the form of variants may be used to predict susceptibility to a phenotype in an unaffected individual or may assist the clinician in predicting the outcomes after the onset of the disease. Both of these are crucial as the former would aid in reducing the future burden of the disease, and the latter would help identify and treat individuals at risk of severe liver disease. In the current review, we summarize the pathogenic mechanisms of ALD and discuss the variants identified to date that may aid in predicting alcohol dependence and the development of cirrhosis in individuals with AUD.

## Introduction

Alcohol consumption historically has been practiced by many as a social engagement and for societal bonding. Though moderate alcohol consumption is associated with cardiovascular benefits, the term “moderate” may not be uniform to each individual (1). Furthermore, consumption of alcohol in excess leads to unpleasant and avoidable adverse outcomes, including an impact on social, occupational, interpersonal, and health. On average, the global consumption of alcohol was reported to be 6.18 liters per person (2). While alcohol use statistics in individuals from the United States of America suggest that 85.6% of people aged 18 or more reportedly drank alcohol at some point in their lifetime, 25.8% and 6.3% reported binge drinking and heavy alcohol use, respectively (3). According to the National Survey of Drug Use and Health (NSDUH) carried out in the year 2019, approximately 14.5 million people aged ≥12 years (9.0 million men and 5.5 million women) were reported to have alcohol use disorder (AUD) (3). The numbers of individuals with AUD are alarmingly high, even among the young aged between 12 and 17 years, where an estimated 4,14,000 (1,63,000 males and 2,51,000 females) are affected (3). However, only 7.2% of the people aged ≥12 years and 6.4% of adolescents who had AUD received treatment.

In 2016, 5.3% of all global deaths translating to 3 million, were attributed to the consumption of alcohol, and alcohol misuse was the seventh leading risk factor for premature death and disability (4, 5). While the World Health Organization (WHO) suggested that alcohol consumption contributed to >200 diseases that ranged from liver diseases, cardiovascular disease, cancer, tuberculosis, and acquired immunodeficiency syndrome (AIDS), the related deaths were 21.3% due to digestive diseases (primarily cirrhosis of the liver and pancreatitis), 19% due to cardiovascular diseases, 12.6% due to cancer and 12.9% due to infectious diseases (6). This puts the economic burden at 249 billion dollars in the United States due to alcohol misuse (7).

Alcohol is the leading cause of liver diseases worldwide (8). However, the number of people affected with liver diseases is disproportionately low compared to a higher number consuming alcohol points to the role of pathomechanics specific to a subset of individuals. Over the years, research points to the overwhelming role of the interactions between host genetics and environment, apart from other triggers for this progression. It is very pertinent to identify the role of genetics in the form of variants and their associated genes not only to understand the pathogenic mechanisms involved in the development of alcoholic liver disease (ALD) at the molecular level but also to explore the putative drug targets for developing advanced therapeutics. This review summarizes the genetics of ALD and variants associated with alcohol dependence.

## Alcoholic liver disease – mechanism of cell injury and pathogenesis

### Alcohol metabolism

Ethanol, a simple alcohol with two carbon atoms, is a small molecule known to diffuse quickly across cell membranes. The absorption of the molecule is influenced by many factors, including age, gender, metabolism, and body weight. Biotransformation of ethanol is predominantly carried out by its oxidation into acetaldehyde, which is the main pathway. The process utilizes nicotinamide adenine dinucleotide (NAD^+^), and is achieved primarily by alcohol dehydrogenase (ADH), which is mainly expressed by the cells of the liver and gastrointestinal tract. Other processes, including catalase and microsomal ethanol oxidation system based on *CYP2E1*, are also involved in ethanol metabolism. Irrespective of the oxidation pathway, ethanol is converted to acetate, a process catalyzed by aldehyde dehydrogenase (ALDH, expressed in cytoplasm and mitochondria). This oxidation and subsequent conversion of ethanol to acetate increase the amount of NADH in the liver. With the enzymes involved in this conversion process predominantly being expressed in the liver, most of the harmful effects of ethanol are, therefore, directly noted in the hepatocytes.

### Oxidative stress response

Accumulation of reactive oxygen species (ROS), predominantly hydrogen peroxide (H_2_O_2_) and superoxide anion (O_2_^-^) concomitant with hypoxia, translocation of bacterial species, and proinflammatory cytokine release is demonstrated to be a direct result of ethanol metabolism. The ROS bind to ethanol/iron atoms to form reactive metabolites (Ferrous oxide: FeO, hydroxyethyl radical: CH_3_CHOH) and are involved in lipid peroxidation of cell membranes. During the pathogenesis of alcohol-induced liver injury, iron is also involved in oxidative stress and is known to promote fibrosis by mediating the catalyzing ROS formation (9). The double-edged cytotoxic effects comprising ethanol metabolism and ROS formation reportedly contribute to apoptosis and necrosis, eventually leading to cell death. Continuous exposure to ethanol is also known to induce glutathione depletion, making the hepatocyte cells more sensitive to oxidative stress (10).

### Edoplasmic reticulum stress

Maturation of proteins, including the folding process, usually occurs in the endoplasmic reticulum (ER). Incomplete maturation of the proteins in response to various stimuli leads to the accumulation of unfolded/misfolded proteins in the ER. This triggers multiple pathways leading to ER stress, which activates NF-kB and JNK, thereby initiating and promoting inflammation. In addition, disruption of calcium homeostasis and activation of pathways, including CHOP-GADD153, triggers apoptosis. Upregulation of aldose reductase (AR) concomitant with an elevation of its metabolites (uric acid, sorbitol, and fructose) in liver specimens was demonstrated in patients with alcohol-associated hepatitis. These events correlated with increased lipid peroxidation byproducts, ER stress, decreased protective ER chaperones, and greater cell death and liver injury (11).

### Genetics of alcoholic liver disease

Gender differences in the progression of ALD with females being more susceptible than males in the background of similar levels of consumption of alcohol, interethnic differences, and higher concordance for cirrhosis in monozygotic twins as compared to dizygotic twins, all point to the role of genetic modifiers for the phenotype. Studies have provided unequivocal evidence in favor of genetic predisposition to organ-specific complications of alcohol consumption. Twin studies in ALD cohorts identified a nearly three-fold higher prevalence in monozygotic compared to dizygotic twins, again pointing to the genetic predisposition to the phenotype (12). Most of the genetic susceptibility identified thus far is predominantly in genes concerning alcohol metabolism and/or those influencing the risk of hepatic damage in individuals with continued alcohol consumption.

### Genetics of alcohol metabolism

The volume, the concentration of ethanol ingested, and the duration of exposure determine the extent of damage to the organs. Metabolism of ethanol involves ADH, ALDH, and CYP2E1 pathways. Multiple ADH and ALDH enzymes are reportedly identified and are encoded by different genes. The details of these genes, including the location, size, number of exons, and the total variants identified thus far, are summarized (**Table 1**). Depending on the type of variants in these genes, the ability to metabolize ethanol varies in individuals and is known to influence an individual’s drinking habits and subsequent risk of alcohol dependence (13). Conversely, certain variants in these genes predispose individuals to a significantly reduced risk of alcohol dependence. In addition, the prevalence of these variants differs among ethnicities confirming the higher risk of ALD associated with particular ethnicities.

**Table 1:** Genes concerning Alcohol Dehydrogenase (ADH) and Aldehyde Dehydrogenase (ALDH).

Official Gene Name	Sequence*	Predominant Expression*	Chromosomal location	Gene Size (bp)**	No. of exons**	Total variants identified**
*ADH1A*	NM_000667.4	Liver	4q23	1454	9	4009
*ADH1B*	NM_000668.6	Liver, fat	4q23	4067	9	4887
*ADH1C*	NM_000669.5	Liver, colon, duodenum, fat, small intestine, stomach	4q23	1454	9	5131
*ADH4*	NM_000670.5	Liver	4q23	2002	9	5287
*ADH5*	NM_000671.4	ALL	4q23	2652	9	4983
*ADH6*	NM_000672.4	Liver	4q23	2802	9	4409
*ADH7*	NM_000673.7	Esophagus	4q23	2119	9	7087
*ALDH1A1*	NM_000689.5	Liver, Duodenum, stomach	9q21.13	2096	13	13847
*ALDH1A2*	NM_009022.4	Adult testis	15q21.3	3386	13	37166
*ALDH1A3*	NM_000693.4	Prostrate	15q26.3	3469	13	10140
*ALDH1B1*	NM_000692.5	Liver, Kidney	9q11.1	3028	2	2224
*ALDH1L1*	NM_001270364.2	Liver, Kidney	3q21.3	3064	23	21277
*ALDH1L2*	NM_001034173.4	Pancreas	12q23.3	7428	23	17591
*ALDH2*	NM_000690.4	Liver, Fat	12q24.2	9561	13	13128
*ALDH3A1*	NM_000691.5	Esophagus, stomach	17p11.2	1639	11	3890
*ALDH3A2*	NM_000382.3	Skin, adrenal	17p11.2	3703	10	7905
*ALDH3B1*	NM_000694.4	Lung, Bone marrow	11q13	2811	10	5943
*ALDH3B2*	NM_001031615.3	Skin, esophagus	11q13	2650	10	5027
*ALDH4A1*	NM_001161504.2	Kidney, liver	1p36	3139	15	9444
*ALDH5A1*	NM_001080.3	Liver, Brain	6p22	5131	10	11753
*ALDH6A1*	NM_001278593.2	Kidney, liver	14q24.3	5462	12	7387
*ALDH7A1*	NM_001182.5	Kidney, liver	5q31	4765	18	15429
*ALDH8A1*	NM_001193480.2	Kidney, liver	6q23.2	2534	7	8612
*ALDH9A1*	NM_000696.4	Fat, thyroid	1q23.1	2395	11	10037
*ALDH16A1*	NM_001145396.2	Spleen duodenum	19q13.33	3099	17	7504
*ALDH18A1*	NM_001017423.2	Duodenum, small intestine	10q24.3	3352	18	13349

*data retrieved from NCBI: Gene ** Data retrieved form ENSEMBL.

### Alcohol metabolism-related genes

Variants in *ADH1B* and *ADH1C* genes are known to lead to the production of enzymes with altered kinetic properties. Three different *ADH1B* alleles have altered amino acids in the sequence that encodes the β-subunit. The amino acid substitutions in both β2 and β3 subunits are known to occur at a position that makes contact with the (NAD^+^) coenzyme that is essential for the oxidation of ethanol. The variants and their associated effect on the metabolism of alcohol are summarized in **Table 2**.

**Table 2:** Summary of variants in Alcohol Dehydrogenase (ADH) and Aldehyde dehydrogenase (ALDH) genes and their effect.

Gene name	Amino acid differences	Protein subunit	Predominant Prevalence	Effect on metabolism
*ADH1A*	None	α	–	Normal
ADH1B*1	Arg48, Arg370	β_1_	**-**	Normal
ADH1B*2	His48, Arg370	β_2_	Asians except Indians, Jewish	Faster oxidation of ethanol
ADH1B*3	Arg48, Cys370	β_3_	African, Native American tribes	Faster oxidation of ethanol
ADH1C*1	Arg272, Ile350	γ_1_	Asians	Faster oxidation of ethanol
ADH1C*2	Gln272, Val350	γ_2_	**-**	Reduced oxidation of ethanol
ADH1C*352Thr	Thr352	**-**	Native Americans	Not known
*ADH4*	Non-coding	π	**-**	Risk for alcoholism
*ADH5*	–	*X*	**-**	**-**
*ADH6*	–	ADH6	**-**	**-**
*ADH7*	–	σ	**-**	**-**
ALDH*2	Glu504Lys	–	Chinese, Japanese and Korean	Reduced/nil oxidation of acetaldehyde. Alcohol flush reaction, nausea, tachycardia. Strongly protective against alcohol dependence

*Gene name.

Two predominant enzymes, ALDH1 encoded by *ALDH1A1* and ALDH2 encoded by *ALDH2*, are known to metabolize acetaldehyde produced during ethanol oxidation. The proteins encoded by these two genes are identical to a more significant extent (70%), including the sequence and structure of genes. The predominant variant in *ALDH2* gene that is extensively worked out is ALDH*2 (**Table 2**). The substitution (Glu504Lys) results in the production of an inactive enzyme; therefore, individuals with this variant cannot oxidize acetaldehyde to acetate. Due to the dominant inheritance, the enzyme may be undetectable even in heterozygous individuals. Individuals with ALDH*2 allele show an alcohol flush reaction even with smaller amounts of alcohol consumption that triggers highly aversive reactions, including severe flushing, tachycardia, and nausea. Studies have reported strong protection against alcohol dependence even with a single copy of the ALDH*2 allele. This beneficial effect is because of the severe adverse effects demonstrated with even small amounts of alcohol consumption. However, environmental interactions are known to modulate the protective effects of this allele, and this was demonstrated by a study where the investigators found an increase of this allele (2.5% to 13% between 1979 and 1992) in Japanese individuals who consumed alcohol (14).

A meta-analysis of 50 association studies of variants reported in genes namely *ADH2, ADH3, CYP2E1*, and *ADLH2* reported significant associations of ADH2*1, ADH3*2, and ALDH2*1 alleles with the risk of alcoholism (15). In addition, subgroup analyses showed an association for ADH2*1 and ADH3*2 only in East Asians and ADH2*1 in Caucasians. Further, the study revealed no significant associations for ADH2*1, ADH3*2, or ALDH2*1 in all subpopulations and no association for the CYP2E1 variant whatsoever. All the alleles were inherited in a dominant mode (15).

A study that genotyped for variants in *ADH2, ADH3, CYP4502E1* (Pst-I and Dra-I), and *ALDH2* in Spaniard alcoholic subjects (without liver disease, with non-cirrhotic liver disease, and with cirrhosis) and compared with non-alcoholic subjects (healthy controls with non-cirrhotic non-alcoholic liver disease and with cirrhosis unrelated to alcohol) reported that variants in the studied genes were not related to alcoholism or susceptibility to alcoholic liver disease in addition to ALDH2 locus being monomorphic (16).

**Figure 1:**
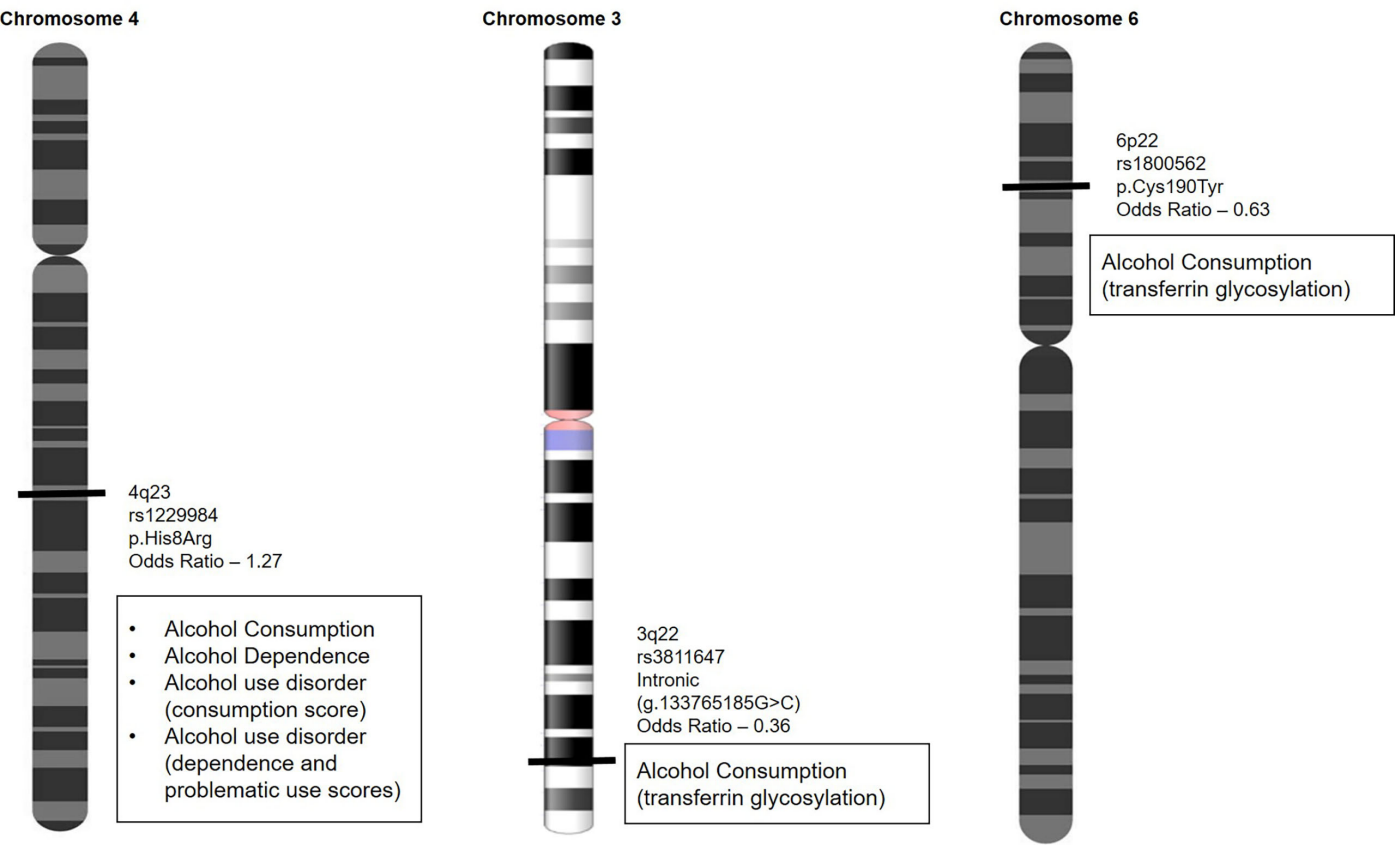
Variants that predict alcohol dependence, alcohol consumption, and alcohol use disorder are used to generate problematic use scores to identify individuals at higher risk of alcohol abuse.

Variants known to predict alcohol sensitivity are currently being investigated by firms that offer variant-based testing directly to consumers under nutrigenetics testing. These firms predominantly test under food sensitivity and include variants that predict alcohol dependence, alcohol consumption, and alcohol use disorder, generating problematic use scores (17). A list of variants that are currently being used is depicted in **Figure 1**.

### Other essential genes associated with ALD

**Figure 2:**
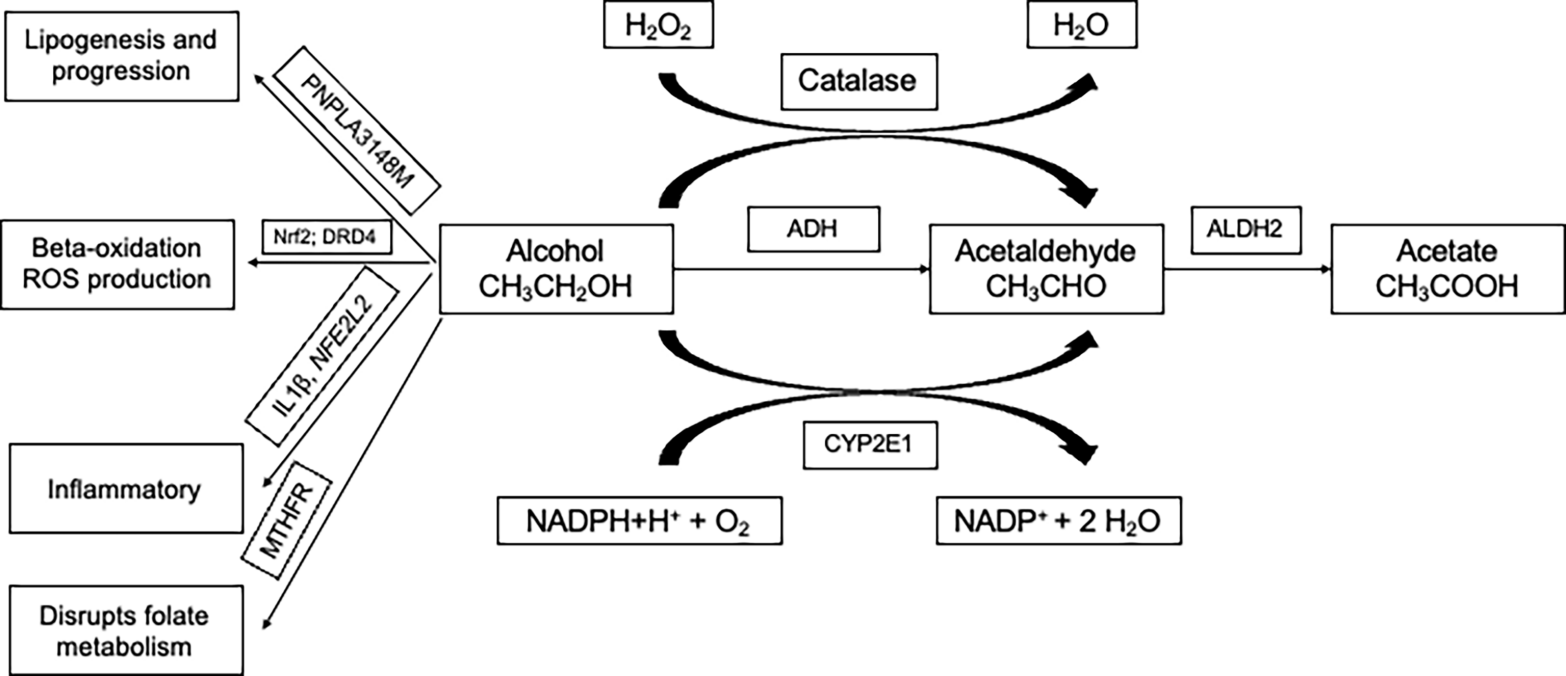
Three main pathways involved in the metabolism of alcohol and involves genes namely ADH, ALDH, and CYP2E1. Multiple ADH and ALDH enzymes are reportedly identified and are encoded by different genes. PNPLA3-148M variant accelerates lipogenesis and progression. Alcohol abuse in the presence of variants in Nrf2 and DRD4 are involved in beta-oxidation and production of ROS (reactive oxygen species). Variants in inflammatory genes are known to modulate alcohol-induced liver injury. Alcohol is known to disrupt the folate cycle involved in the metabolism of methionine, which is crucial in providing methyl groups required for the methylation of DNA. ADH, Alcohol dehydrogenase; CYP2E1, cytochrome P450 2E1 subfamily; ALDH, Aldehyde dehydrogenase; PNPLA3, Patatin-like phospholipase domain-containing protein 3; IL1B - Interleukin 1 beta; NFE2L2 - NFE2 Like BZIP Transcription Factor 2; DRD2, Dopamine receptor D2; MTHFR, Methylenetetrahydrofolate reductase; NADPH, Nicotinamide adenine dinucleotide phosphate; H_2_O_2_, Hydrogen peroxide; H_2_O, Water; O_2_, Oxygen.

Certain loci confer a higher risk of ALD (18). Most prominent among them are variants in genes of *methylenetetrahydrofolate reductase (MTHFR), interleukin 1β (IL1B)*, *NFE2L2, RASGRF2*, *CYP2E1, DRD2, TM6SF2, MBOAT7*, and *PNPLA3* that were reportedly associated with the phenotype. The summary of information on the genes (**Table 3**) and their mechanism (**Figure 2**) is given. A detailed description of the role of these genes in ALD is discussed below.

**Table 3:** Summary of information on genes associated with Alcoholic Liver Disease (ALD).

Gene Name (Symbol)	Chromosomal location	NCBI Gene ID	Size in bp (No. of Exons)	No. of Amino acids	Function	Variants associated with ALD
Methylenetetrahydrofolate reductase (MTHFR)	1p36.22	4524	7018 (12)	656	The gene encodes an enzyme called methylenetetrahydrofolate reductase. This enzyme converts 5,10-methylenetetrahydrofolate to 5-methyltetrahydrofolate, a primary form of folate found in blood. It is is necessary for the conversion of homocysteine to methionine.	rs1801133 rs1801131
Interleukin 1 beta (*IL1B)*	2q14.1	3553	1507 (7)	269	The protein encoded by this gene is a member of the interleukin 1 cytokine family. This cytokine is an important mediator of the inflammatory response.	-511C rs16944
NFE2 Like BZIP Transcription Factor 2 (*NFE2L2)*	2q31.2	4780	2446 (5)	605	The gene encodes a transcription factor and is a member of a small family of basic leucine zipper (bZIP) proteins.It is known to regulate genes that encode proteins involved in response to injury and inflammation which includes the production of free radicals.	rs35652124
Ras Protein Specific Guanine Nucleotide Releasing Factor 2 (*RASGRF2)*	5q14.1	5924	8482 (27)	1237	The gene encodes a calcium-regulated nucleotide exchange factor activating both RAS and RAS-related protein, RAC1, thereby, coordinating the signaling of distinct mitogen-activated protein kinase pathways.	rs26907 rs61764370
Cytochrome P450 2E1 gene (*CYP2E1)*	10q26.3	1571	1674 (9)	493	The gene product is a monooxygenase which catalyzes reactions involved in drug metabolism and synthesis of cholesterol, steroids and other lipids. This protein localizes to the endoplasmic reticulum and is induced by ethanol.	c.-1053C>T rs2031920
Dopamine receptor D2 (DRD2)	11q23.2	1813	2808(8)	443	The gene encodes the D2 subtype of the dopamine receptor. This G-protein coupled receptor inhibits adenylyl cyclase activity.	-141C Ins/Del, (rs1799732) TaqI A TaqI B
Transmembrane 6 superfamily member 2 (*TM6SF2)*	19p13.11	53345	1518 (10)	377	The protein coded by this gene is involved in regulation of lipid metabolic process	rs58542926
Membrane Bound O-Acyltransferase Domain Containing 7 *(MBOAT7)*	19q13.42	79143	2294(8)	472	This gene encodes a member of the membrane-bound O-acyltransferases family of integral membrane proteins that have acyltransferase activity. The encoded protein is a lysophosphatidylinositol acyltransferase that has specificity for arachidonoyl-CoA as an acyl donor. This protein is involved in the reacylation of phospholipids.	rs641738
Patatin-like phospholipase domain-containing protein 3 (*PNPLA3)*	22q13.31	80339	2753(9)	481	The protein encoded by this gene is a triacylglycerol lipase. It mediates triacylglycerol hydrolysis in the adipocytes. It may also be involved in the balance of energy usage/storage in adipocytes.	rs738409

## Genetic modifiers of alcoholicliver disease

### Transmembrane 6 superfamily member 2

The protein coded by the gene TM6SF2 is localized to the ER membrane and intermediate compartment membrane and is involved in regulating the lipid metabolic process. A variant in this gene (p.Glu167Lys; rs58542926) was initially identified by an exome-wide screening of variants for non-alcoholic fatty liver disease (NAFLD) (19). Subsequent to the identification, the variant was demonstrated to regulate liver fat metabolism by influencing triglyceride secretion and hepatic lipid droplet content (20). In addition, it has been shown to induce lipid biosynthesis and reduce the secretion of Apolipoprotein B (21). A genome-wide association study for alcohol-related cirrhosis in individuals of European descent identified this variant in *TM6SF2* apart from *MBOAT7* as new risk loci (22). A similar alcohol-related cirrhosis risk for the variant was reported in the British and Irish populations (23). However, few studies could not replicate the association. A study that explored the association of this variant in TM6SF2 gene apart from other variants with the risk of hepatic fibrosis or liver cirrhosis of different etiologies suggested that the variant was not associated with hepatic fibrosis, liver cirrhosis induced by alcohol in an Eastern European population (24).

### Membrane-bound O-acyltransferase domain containing 7

This gene encodes a lysophosphatidylinositol acyltransferase protein and has an affinity for arachidonoyl-CoA as an acyl donor. It is reportedly involved in the reacylation of phospholipids as part of the phospholipid remodeling pathway, also known as the Land cycle. A genome-wide association study that followed up with fine mapping identified a variant (rs641738) in *MBOAT7* gene to be associated with alcohol-related cirrhosis (22). Later, the variant was also reported to be associated with an increased risk of NAFLD in individuals of European descent. However, other studies have reported a negative association of the variant (rs641738) in MBOAT7 with hepatic fibrosis, alcohol, or hepatitis C-related liver cirrhosis in an Eastern European population (24, 25). Although there is unequivocal evidence for the association of the variant with liver fat, ALT, and fibrosis in NAFLD, a similar association with ALD is lacking (26). Further studies involving a larger multi-ethnic sample are warranted before arriving at a definite conclusion.

### Patatin-like phospholipase domain-containing protein 3

The protein encoded by the gene PNPLA3 is a lipid droplet-associated protein that is demonstrated to have triglyceride and retinyl esters hydrolase activity. In humans, this gene is highly expressed in the liver and moderately in the brain, kidney, adipose, and skin (27, 28). A genome-wide association study (GWAS) in a multiethnic population comprising Hispanic, African American, and European American individuals identified a variant in this gene associated with hepatic fat concentration for the first time. The variant (rs738409; C>G) is a cytosine to guanine change that causes methionine to isoleucine conversion at the 148-amino acid position (I148M) (29). Following this discovery, multiple studies have associated this variant with the risk of ALD and elevated liver enzymes (30, 31). A cross-sectional study on the US population explored the interaction between alcohol consumption and *PNPLA3* variant (rs738409) (32). The results from this study suggested that the variant conferred a strong genetic susceptibility to liver disease and was modifiable by the level of alcohol consumption. They further reiterated that keeping the consumption of alcohol low may offset the genetic predisposition to liver disease in variant carriers (32). Another vital study demonstrated that although the variant (rs738409) in the PNPLA3 gene is associated with ALD, it did not correlate with serum IL6, IL10, IL8, and CCL2 levels in the Russian population (33).

A GWAS in patients with alcohol-related cirrhosis of European ethnicity found a genome-wide significant risk association of rs738409 in *PNPLA3* (Odds ratio = 2.19; G allele) and rs4607179 near *HSD17B13* (Odds ratio = 0.57; C allele. In addition, the study identified a protective association at rs374702773 in Fas-associated factor family member 2 (FAF2; Odds ratio = 0.61; del(T) allele) for alcohol-related cirrhosis when conditional analysis accounting for the *PNPLA3* and *HSD17B13* loci was carried out (34). A GWAS was carried out in the Korean Genome and Epidemiology Study Health Examination cohort data to assess the risk of developing ALD in nondrinkers, light drinkers, and heavy drinkers. The study reported an association of loci, namely gamma-glutamyltransferase 1 (GGT1; rs2006227), zinc protein finger 827 (*ZNF827*; rs1183910), and HNF1 homeobox A (*HNF1A*; rs1183910) with ALD risk, in addition to 5 variants on Chromosome 11 that showed protective effects against ALD (35).

## Modifiers of cytokine activity and inflammation

### Interleukin 1 beta

Interleukin 1β apart from interleukin 1α is known to bind to the same receptor IL-1R type 1. They have similar functions and are involved in proinflammatory responses (36). The levels of IL-1β in the serum are high in patients with ALD, especially those with cirrhosis. A study carried out on East Indians suggested that variants in genes, including IL-1β, modulate the indices of alcohol-induced liver injury. The study identified that a variant (-511C) in the promoter region of the *IL-1β* gene was significantly overrepresented in ALD patients compared to individuals who misused alcohol without liver disease (controls). They also suggested that the risk genotype (-511CC) influenced the total levels of bilirubin, albumin, and alanine transaminase among alcohol users (37). Significantly higher occurrence of variants in IL-1 β gene in Japanese heavy drinkers who progressed to ALD-cirrhosis also strengthens the suspicion of its role in ALD (38). However, a meta-analysis reported no significant association between variants in *IL*-*1β* (−* 511C* >* T* and +* 3953T* >* C*) and ALD susceptibility. The meta-analysis included a total of 825 ALD patients and 743 controls that were pooled from 10 studies (39). These results point to the role of ethnicity-specific susceptibility and needs to be explored further. In addition, the role of variants in IL-1β both in conferring susceptibility and/or progression of ALD needs to be explored in a large multi-ethnic population.

### NFE2 Like BZIP transcription factor 2

NFE2 Like BZIP Transcription Factor 2 *(NFE2L2)* codes for transcription factor nuclear factor erythroid-related factor 2 (Nrf2). Nrf2 is known to regulate the expression of proteins that protect against inflammation and oxidative stress caused by excess consumption of alcohol and therefore is considered a potential candidate marker for ALD susceptibility. A study that explored the association of variants rs35652124, rs4893819, and rs6721961 in *NFE2L2* gene identified that the variant rs35652124 predisposed individuals to ALD. Hematoxylin & eosin and immunohistochemistry of Nrf2 staining suggested that the variant was associated with a lower level of Nrf2 expression. In addition, liver tissues from ALD patients with this variant were associated with severe inflammatory activity (40).

## Modifiers of neurotransmission

### Ras protein specific guanine nucleotide releasing factor 2

Variants in the gene have been associated with various diseases, including alcohol-related disorders, predominantly through their involvement in neurotransmission and inflammation (41). A variant (rs26907) in the Ras-specific guanine nucleotide-releasing factor 2 gene (*RASGRF2*) was associated with higher alcohol consumption and also variability in alcohol-induced reward response (42). These findings added credence to the role of the gene in alcohol misuse susceptibility. This role is further reiterated by its regulation by RasGRF2 in synaptic transmission and its putative role in inflammation-related pathways *via* the activation of MAPKs (mitogen-activated protein kinases) and ERKs (extracellular signal-regulated kinases) (43–45). Other important variants also have been reported for their potential association with alcoholism. A variant (rs61764370) in *KRAS* gene is promising as it is located in the miRNA binding site for the member of the *let-7* family. Individuals with the G allele at this position have impaired binding of let-7 and may be relevant in alcoholism, as a study showed the relevance of K-ras activity and alcohol craving in an animal model (46). Furthermore, let-7 is shown to be overexpressed in the brains of alcohol abusers and has been associated with an inflammatory response triggered by alcohol consumption (47). Also, the regulatory functions of let-7 are demonstrated to affect the activation of stellate cells in the liver and are involved in liver injury (48).

### Dopamine receptor D2

The central dopaminergic system is known to influence and regulate brain reward mechanisms and is considered to play a critical role in the development of dependence on a range of psychoactive substances, including ethanol (49–53). Alcohol reportedly stimulates dopamine receptors, promoting its release in the ventral striatum, activating the mechanism involving craving and incentive salience attributions paving the way for increased ethanol consumption (54). Therefore, studying the role of variants in genes concerning dopaminergic pathways in alcohol dependence is relevant. Various studies focused on association studies of the D1-D5 dopamine receptors and transporter protein (DAT) have identified the relevance of *DRD2* gene in susceptibility to alcohol dependence. Although many studies focussed on exploring the role of DRD2 variants in alcohol dependence, the findings have primarily been controversial. Most of the significant associations of variants in the gene have been reported from the European/European American ethnicities, with studies in the Taiwanese population (Atayal natives) registering a negative association (55). The most commonly investigated variants in the *DRD2* gene are -141C Ins/Del, TaqI B, and TaqI A, which are associated with alcohol dependence (56, 57). Insertion of the nucleotide “C” in the promoter region of the DRD2 gene at the -141 position (rs1799732) is reported to be associated with receptor density (58). As the TaqI B variant is in the 5`untranslated region (5` UTR), it is predicted to play an essential role in transcriptional regulation (59). The TaqI A variant is associated with low levels of the availability of D2 dopamine receptors in the striatum (60). Although this variant was initially reported to be in the 3`UTR region of the DRD2 gene, it is now identified that the variant is located in the gene *ANKK1 (*ankyrin repeat and kinase domain containing), where it is reportedly a missense variant. The variant in *ANKK1* gene is associated with increased activity of the final enzyme named aromatic L-amino acid decarboxylase, which is involved in the biosynthesis of dopamine (61). A study from India that explored the association of these three variants in their association with alcohol dependence concluded that two variants (-141C Ins allele and TaqI A1) were associated with alcohol dependence (62).

### Cytochrome P450 2E1 gene

This gene belongs to a member of the cytochrome P450 superfamily of enzymes that catalyze many reactions, including drug metabolism, synthesis of cholesterol, and lipids, and being induced by ethanol. The enzyme is involved in various processes, such as gluconeogenesis, and is implicated in liver cirrhosis (63). Functionally relevant variants in the *CYP2E1* gene are therefore crucial in susceptibility to ALD. A study that explored the genetic polymorphisms in the 5`-flanking region of the *CYP2E1* with the transcriptional regulation of the gene reported that polymorphisms affect the transcriptional regulation and may lead to inter-individual variation (64). A study that did not find any association between *CYP2E1* c.-1053C>T variant with susceptibility to alcohol dependence reported that it might influence the expression of obsessive-compulsive and anxiety symptoms (65). A family-based genome-wide linkage analysis involving sibling pairs reported that linkage analysis detected significant linkage to *CYP2E1* gene but was diminished. The authors suggested that variants in and around *CYP2E1* affect the level of response to alcohol consumption, thus providing evidence for its role in alcohol addiction (66). A study reported no association of variants (CYP2E1*1D, *5B, *6, and *1B) with alcohol dependence in the Han Taiwanese population (67).

## Other important genes

### Methylenetetrahydrofolate reductase

This gene is involved in the conversion of folate to methionine. Specifically, this enzyme is required to convert 5,10 methylenetetrahydrofolate to 5-methyltetrahydrofolate, the primary circulatory form. This circulatory form of 5-methyltetrahydrofolate is necessary for the subsequent multistep conversion of homocysteine to methionine. In animal models, folate deficiency was shown to disturb hepatic methionine metabolism, thereby promoting liver injury in ethanol-fed micropigs (68). A variant at the 677 position (rs1801133; C677T; A222V) in the human *MTHFR* gene is associated with reduced protein and increased homocysteine levels. The variant is associated with multiple diseases, including hepatic steatosis, cirrhosis, and hepatocellular carcinoma, predominantly due to higher homocysteine levels. Another variant, A1298C (rs1801131), in this gene, is reported to be in high linkage disequilibrium with the C677T and is also clinically relevant. However, a recent meta-analysis examining the association between the C677T variant in the *MTHFR* gene and the risk of alcohol dependence confirmed that there was no association (69). In addition, the study concluded that there was no association between the variant and alcohol dependence in Asians and Caucasians on sub-group analysis (69).

## Other risk factors for alcoholic liver disease

### Gender

Women are at a higher risk of ALD and acute liver failure as compared to males (70, 71). It is known that for a given dose of alcohol, women are more susceptible to toxic effects as compared to males, although the latter is known to abuse alcohol more than the former. A population-based 13-year prospective study carried out in Denmark that followed 13,000 participants demonstrated that the risk of developing ALD in women was higher as compared to the males, even with higher consumption of alcohol in the males [7 to 13 beverages per week (84-156 g) in females Vs. 14 to 27 beverages per week (168-324 g) in males] (70). In addition, a higher relative risk of alcohol-induced cirrhosis was noted in women as compared to men [7.0 (95% CI, 3.8-12.8) Vs. 17.0 (95% CI, 6.8-40.8)], although it is less frequently diagnosed in women (72).Experiments in animal models demonstrate that this effect is due to relatively higher endotoxin levels and increased gut permeability, variability in the concentrations of estrogen receptors in the liver, and activation of Kupffer cells by estrogen. All these lead to increased inflammation and necrosis. In addition, alcohol induces differential expression of genes responsible for higher oxidative stress, inflammation, and injury in female rats (73). Therefore, females have an increased risk of developing ALD even with small amounts of alcohol consumed.

### Ethnicity

Substantial ethnicity-based differences in hospitalizations and mortality have been reported for patients with ALD. A study that assessed the liver and alcohol-related hospitalizations and deaths in ALD and specific alcohol-related diseases (ARD) reported ethnicity-based differences in the population. While Indians had a 75% higher risk for men, White British and Pakistani men had a relatively lower risk of ALD. A 2-fold higher risk for ARD was noted for White Irish men and any mixed background women, lower risk was noted for Pakistani and Chinese men and women (74). ARD included diseases attributed to alcohol: liver disease, chronic pancreatitis, poisoning, mental and behavioural disorders, nervous system disorders, cardiomyopathy, and gastritis. In addition, Hispanics present at 4-10 years younger age for ALD than White/Caucasian patients (75).

### Nutritional status

The most frequent complication of ALD is malnutrition. The reason for malnutrition is increased resting energy expenditure, limited ingestion or improper absorption, digestion, and utilization of nutrients due to the effect of alcohol metabolism (76, 77). The relationship between alcoholism and malnutrition is biderctional. Loss of muscle mass of the skeleton or sarcopenia and disturbances in energy metabolism are the two major known components of malnutrition in liver disease. Although it is known that alcohol is metabolized directly in the skeletal muscle contributing to the loss of muscle mass, however, its contribution to other organ injuries is currently unknown. Duration and the use/abuse of alcohol, quantity consumed, and other underlying causes/severity of liver disease are few of the factors that affect the severity of malnutrition in ALD (78). Furthermore, patients with ALD frequently are known to have deficiencies in proteins and vitamins that contribute to the severity. The highly reactive toxic metabolites generated by alcohol metabolism, including acetaldehyde and/or oxygen-containing molecules, interfere with the metabolism of other nutrients, primarily lipids, thereby contributing to the damage of the hepatic cell. Although a number of clinical trials and meta-analyses have been reported in the literature on nutritional support in both cirrhosis and ALD, they have not been conclusive for various reasons (79, 80). Therefore, a balanced diet can compensate for the general existence of malnutrition. In general, a daily intake of about 2000kcal comprising ~1.5g/kg/d protein may generally benefit patients with ALD (81). The intake may be distributed over the day giving short intervals between meals with an appropriate route of administration (78).

### Comorbidities and ALD

It is well recognized that the presence of comorbidities contributes to the progression of ALD. The most common comorbidities at diagnosis of cirrhosis were predominantly arterial hypertension, type 2 diabetes, and obesity (82). The presence of diabetes increases the risk for cirrhosis and hepatocellular carcinoma among patients with both ALD and non-alcoholic fatty liver disease (82, 83). A large population-based study reported that age, male sex, obesity, and components of metabolic syndrome (MetS) were independent predictors of liver-related mortality in ALD patients (84). Diabetes is a significant predictor for severe liver disease and development of cirrhosis among patients who misuse alcohol (85, 86).

### Tools and techniques to identify variants

Advances in genomics over the years has enabled us to probe the genome to understand its role in the pathogenesis of the disease. Alterations in the genome are majorly being used to predict the susceptibility to a phenotype/clinical condition or the risk of progression after the onset of the disease. Alterations in the genome may be at the single nucleotide level (variants) or large microscopically visible chromosomal aberrations. The variants can occur in the coding and non-coding regions of the genome and may be classified as Single nucleotide variants (SNV), multi-nucleotide variants (MNV), or indels (Insertions/deletions) depending on the number of nucleotides that are changed. Copy number variants (CNVs) that include inversions, translocations, deletions, duplications, and insertions are large, typically greater than 50 base pair rearrangements in the genome. Karyotype analysis by GTG banding is the preferred technique for detecting chromosomal aberrations (>5 Mb). Other advanced molecular techniques, including fluorescent *in-situ* hybridization (FISH) and microarray-based comparative genomic hybridization (aCGH) are routinely applied for a more accurate analysis. However, these platforms do not detect mosaicism, balanced translocations, and inversions. Other relevant molecular biology techniques including multiplex ligation-dependent probe amplification (MLPA) may also be used. Restriction Fragment Length Polymorphism (RFLP), Allele-specific PCR (Amplification-refractory mutation system (ARMS)-PCR), Probe based detection (Taqman probes) on Real time PCR (qRT-PCR) are the choice of methods for detecting single nucleotide variants that have prior susceptibility information. DNA microarray, DNA sequencing (Sangers sequencing and Next generation sequencing) are powerful techniques to map variants at the genome level with the disease and are predominantly employed by the Genome-wide association studies that are considered an unbiased way of identifying genetic susceptibility.

### Alcohol and hepatocellular carcinoma

Progression to hepatocellular Carcinoma (HCC) is seen in patients with underlying infections (hepatitis B and C) and metabolic disease (non-alcoholic steatohepatitis [NASH]) apart from ALD. While the incidence of HBV/HCV-related HCC has declined with the introduction of newer therapies, a rise in cases of HCC associated with NASH and ALD is seen (87–89). Although non-cirrhotic HCC is common in NAFLD and viral hepatitis, the progression to HCC in cases with underlying NASH/ALD is typically after steatohepatitis, fibrosis, and cirrhosis (90). A study that characterized the role of IL-17A (a tumour-promoting cytokine) and its receptor IL-17RA signaling in the pathogenesis of HCC in an animal model and human specimens demonstrated that global deletion of IL17-RA suppressed HCC in an alcohol-fed animal model. They suggested that IL-17A may be a potential therapeutic target for patients with alcohol-induced HCC (91). Although it was known that individuals who consume alcohol and are heterozygous for ALDH2*1/*2 or homozygous for ALDH2*2/*2 variants have ALDH2 deficiency and are at a higher risk of developing digestive tract cancers, the mechanism for the same was unknown (92). An *in vivo* and *in vitro* mechanistic study indicated that Aldh2-deficient hepatocytes produced a large amount of harmful oxidized mitochondrial DNA *via* the extracellular vesicles after CCL4 plus ethanol exposure. These were then transferred into the neighboring HCC cells and, along with acetaldehyde, activated multiple oncogenic pathways, including the JNK, STAT3, BCL-2, and TAZ, thereby promoting HCC (93).

### Polygenic risk scores and ALD

Risk stratification scores predominantly based on susceptibility conferring genetic loci were developed primarily to identify individuals with alcohol consumption who may progress to develop cirrhosis/HCC. A study compared three variants, *PNPLA3*:rs738409, *SUGP1-TM6SF2*:rs10401969, and *HSD17B13*:rs6834314, between cases with alcohol-related cirrhosis and controls with a history of alcohol consumption but no evidence of liver disease. The study concluded that stratification of heavy drinkers is possible based on the three variant risk scores and diabetes status, and early preventative interventions might benefit the patient (86).

### Importance of genetic factors for the management of ALD

Understanding the genetic susceptibility both in asymptomatic individuals who are at a higher risk to develop ALD as well as patients with ALD with a risk of progression would be beneficial to reduce the burden of the disease on the healthcare system. Genotyping for variants with an ability to predict alcohol dependence in an individual and assessing the risk will aid in counselling the individual to curtail alcohol consumption. Variants may be routinely genotyped in ALD patients to assess the genotype and the risk of progression with an intention of treating them aggressively. Identifying additional variants by employing whole exome sequencing at the genome level that is associated with susceptibility and risk of progression would aid in developing personalized programs for prevention and/or treatment. Additionally, novel gene targets from the data may be identified and explored as potential therapeutic targets. Currently genotping of variant in *PNPLA3* gene is routinely being used by the clinicians to assess the risk. With access to Next generation sequening and routine use of whole exome and clinical exome sequencing in clinical practice, in furture, polygenic risk scores and algorithms that integrate environmental risks with genetic data would aid the clinician in accurate prediction of onset in high-risk individuals and the risk of progression.

## Conclusion

**Figure 3:**
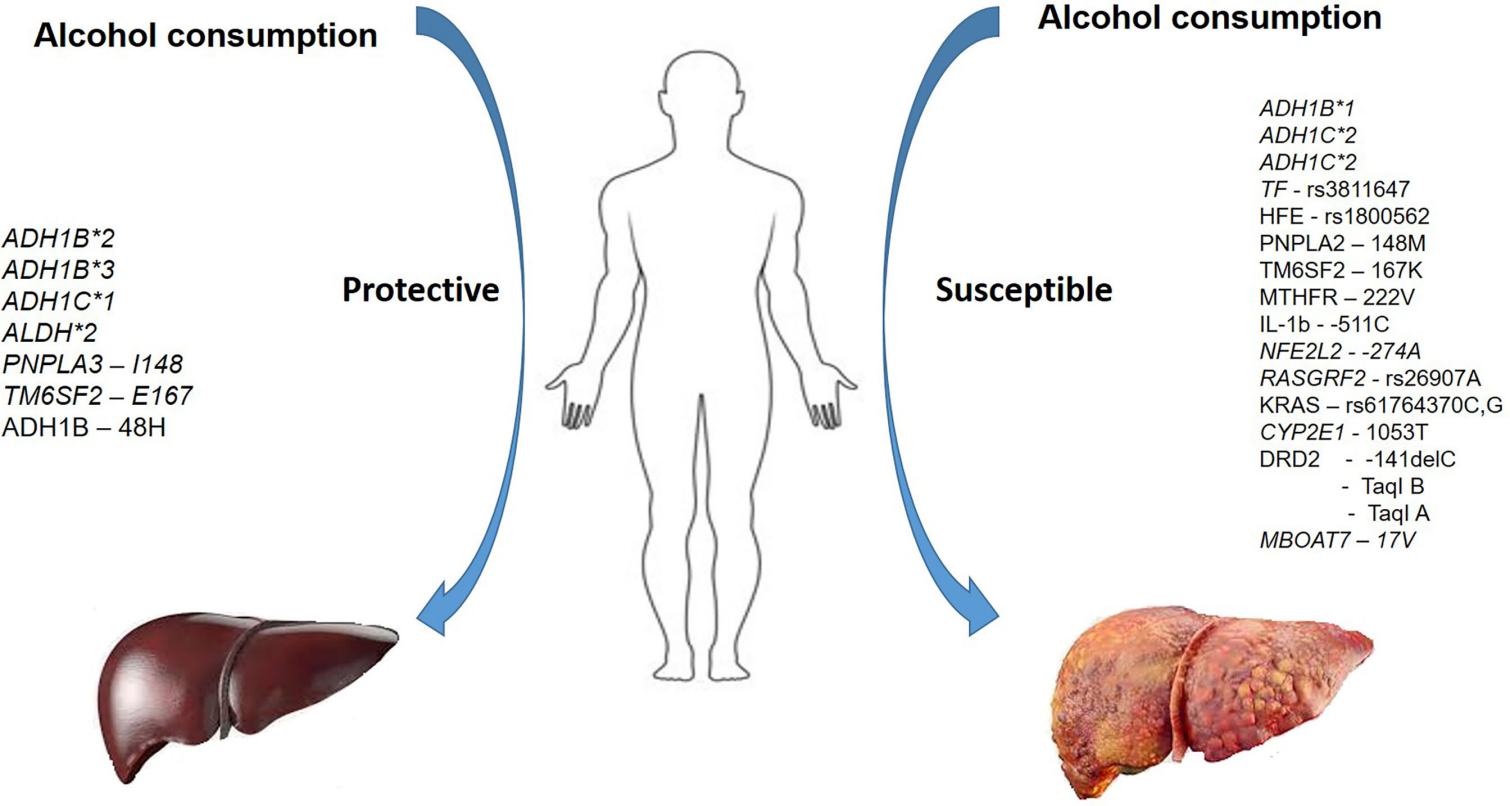
A summary of variants in various genes conferring protection and susceptibility in the presence of alcohol use.

Although higher numbers of individuals reportedly consume alcohol, a fraction of individuals progresses to develop ALD and cirrhosis. ALD is a complex, multi-factorial disease where interactions between genetic susceptibility and the environment precipitate the phenotype. While a compelling role for a few variants across ethnicities is reported (PNPLA3), conflicting results are reported for other genes. Identifying the comprehensive genetic susceptibility to the risk of developing ALD (**Figure 3**) and developing algorithms that aid in identifying individuals at-risk of ALD may be beneficial in reducing the burden of the disease. In addition, routing genotyping of risk variants predictive of developing the severe disease as part of diagnostic workup aids in identifying this subset of patients who may require aggressive treatment.

## Author contributions

AK and RV made the study concept and design. Figures by MS. RV prepared the initial draft. AK, PR, and DR critically edited and supervised the project. All authors contributed to the article and approved the submitted version.

## Conflict of interest

The authors declare that the research was conducted in the absence of any commercial or financial relationships that could be construed as a potential conflict of interest.

## Publisher’s note

All claims expressed in this article are solely those of the authors and do not necessarily represent those of their affiliated organizations, or those of the publisher, the editors and the reviewers. Any product that may be evaluated in this article, or claim that may be made by its manufacturer, is not guaranteed or endorsed by the publisher.
